# The influences of morphine or ketamine pre-treatment on hemodynamic, acid-base status, biochemical markers of brain damage and early survival in rats after asphyxial cardiac arrest

**DOI:** 10.1186/s12871-019-0884-6

**Published:** 2019-11-20

**Authors:** Vladimir Kuklin, Nurlan Akhatov, Timofei Kondratiev, Aidos Konkayev, Abai Baigenzhin, Maiya Konkayeva, Temirlan Karibekov, Nicholas Barlow, Torkjel Tveita, Vegard Dahl

**Affiliations:** 1 Department of Anaesthesiology and Intensive Care Medicine, Akershus university hospital, Sykehusveien, 25, 1478 Lørenskog, Norway; 2 Department of Anaesthesiology and Intensive Care Medicine, Astana Medical University, Nur-Sultan, Kazakhstan; 3 Department of Anaesthesiology and Intensive Care Medicine, National Scientific Medical Center, Nur-Sultan, Kazakhstan; 4 Anaesthesia and Critical Care Research Group, Department of Clinical Medicine, UiT – The Arctic University of Norway, 9037 Tromsø, Norway; 5 Division of Surgical Medicine and Intensive Care, University Hospital of Northern Norway, 9038 Tromsø, Norway; 6 Department of Anaesthesiology and Intensive Care Medicine, University of Oslo, Oslo, Norway

**Keywords:** Morphine, Ketamine, Rats, Asphyxial cardiac arrest, Early survival

## Abstract

**Background:**

In different models of hypoxia, blockade of opioid or N-methyl-D-aspartate (NMDA) receptors shows cardio- and neuroprotective effects with a consequent increase in animal survival. The aim of the study was to investigate effects of pre-treatment with Morphine or Ketamine on hemodynamic, acid-base status, early survival, and biochemical markers of brain damage in a rat model of asphyxial cardiac arrest (ACA).

**Methods:**

Under anaesthesia with Thiopental Sodium 60 mg/kg, i.p., Wistar rats (*n* = 42) were tracheostomized and catheters were inserted in a femoral vein and artery. After randomization, the rats were pre-treated with: Morphine 5 mg/kg i.v. (*n* = 14); Ketamine 40 mg/kg i.v. (n = 14); or equal volume of i.v. NaCl 0.9% as a Control (n = 14). ACA was induced by corking of the tracheal tube for 8 min, and defined as a mean arterial pressure (MAP) < 20 mmHg. Resuscitation was started at 5 min after cardiac arrest (CA). Invasive MAP was recorded during experiments. Arterial pH and blood gases were sampled at baseline (BL) and 10 min after CA. At the end of experiments, all surviving rats were euthanised, brain and blood samples for measurement of Neuron Specific Enolase (NSE), s100 calcium binding protein B (s100B) and Caspase-3 (CS-3) were retrieved.

**Results:**

At BL no differences between groups were found in hemodynamic or acid-base status. After 3 min of asphyxia, all animals had cardiac arrest (CA). Return of spontaneous circulation (MAP > 60 mmHg) was achieved in all animals within 3 min after CA. At the end of the experiment, the Ketamine pre-treated group had increased survival (13 of 14; 93%) compared to the Control (7 of 14; 50%) and Morphine (10 of 14; 72%) groups (*p* = 0.035). Biochemical analysis of plasma concentration of NSE and s100B as well as an analysis of CS-3 levels in the brain tissue did not reveal any differences between the study groups.

**Conclusion:**

In rats after ACA, pre-treatment with Morphine or Ketamine did not have any significant influence on hemodynamic and biochemical markers of brain damage. However, significantly better pH level and increased early survival were found in the Ketamine pre-treated group.

## Background

Almost 35 years ago, Dr. Peter Safar wrote that “cerebral recovery from more than 5 min of cardiac arrest is hampered by complex secondary derangements of multiple organ systems after reperfusion” [1]. Actually, these 5 “golden” minutes determine the ability of cerebral neurones to regain ordinary function after anoxia. The ordinary function of cerebral neurones is conduction of electrical impulses across their length from the post-synaptic membrane of dendrites to the presynaptic membrane of an axon. The process is based on exchange of Ca^2+^, Na^+^ and K^+^ between the extra- and intracellular space of cerebral neurones, and therefore a lot of energy in the form of adenosine triphosphate (ATP) is needed to remove Ca^2+^ and Na^+^ from the intracellular space of these cells. Cardiac arrest (CA) initiates a switch to anaerobic metabolism with very low production of ATP [2] and the increased [2] levels of lactate and H+. Both acidosis and the lack of ATP inhibit the ions pumps, which are responsible for handling excessive intracellular accumulation of Ca^2+^ and Na^+^ [2]. Moreover, preclinical studies demonstrate that acute hypoxia results in an uncontrolled release of glutamate with consequent stimulation of the N-methyl-D-aspartate (NMDA) receptors causing an excessive Ca^2+^ influx [3–8]. Meanwhile, the ATP reservoir in neurones can be completely depleted after 5 min of no-flow state. In case of oxygen supplying restoration, two molecules of ATP are initially required to split glucose and restart the cellular respiration. Thus, the presence or absence of these two molecules of ATP in neurones actually determines restoring of both oxidative phosphorylation and the ordinary function of the neurones. Finally, prolonged intracellular Ca^2+^ overload results in increased mitochondrial permeability causing following release of cytochrome C from mitochondria, and consequent cleavage and activation of caspase-3 [9, 10]. Caspase-3 is an essential protease, which is involved in the early stage of apoptosis and it is generally accepted as a hallmark of irreversible cell death [10]. Recently, early elevated blood levels of two specific biochemical markers of neuronal damage, namely neuron-specific enolase (NSE) and S-100B protein, were also found to be associated with illness severity on hospital arrival, and with poor outcome after cardiac arrest [11]. Today, only therapeutic hypothermia has been shown to have a beneficial impact on the ion pump dysfunction, and thereby reduce neurotoxicity [12]. Interestingly, in hibernators, hypothermia is also believed to protect against hypoxic brain damage [13]. Meanwhile, if naloxone, a non-selective opioid receptor antagonist, is injected during the maintenance phase of hibernation, arousal is quickly achieved and the protective effects will vanish [13]. Delta opioid peptides, previously discovered to induce hibernation, have also been shown to protect rats from hypoxic brain damage [14]. Based on the ability of opioids to reduce the level of cyclic adenosine monophosphate (cAMP), and consequently to block Na^+^ channels, it would be logic to propose that opioids might prevent the disturbance of ionic homeostasis during acute hypoxia. Indeed, preclinical studies demonstrate that pre-treatment with opioids can preserve cellular integrity after acute hypoxia in many organs and tissues including: intestine [15], skeletal muscle [16], myocardium [17, 18] and brain [19, 20]. Moreover, Morphine has been shown to significantly increase the early survival of mice and rats after acute hypoxia condition [21, 22]. Opioid receptor agonists also have demonstrated to cause increased tissue preservation and survival time of organs before their use in transplantation surgery [23]. In addition, high doses of opioids have been shown to inhibit NMDA receptors [24]. Other experimental studies have shown that inhibition of the NMDA receptor by Ketamine may reduce neuronal apoptosis and attenuate the systemic inflammatory response to tissue injury [25–27]. Moreover, the sympathomimetic effects of Ketamine might help to facilitate recovery of systemic blood pressure during cardiopulmonary resuscitation (CPR) [28]. All anaesthetics, with their ability to antagonise glutamate-mediated excitotoxicity and inflammation might be the logic candidates for neuroprotective treatment during cardiac arrest. However, the additional ability of most anaesthetics to produce vasodilatation with a significant reduction of blood pressure can be the main argument against the idea to test their effects during CPR in humans. Theoretically, due to their minimal influences on hemodynamic status, Ketamine as well as Morphine might be considered as the safe candidates during neuroprotective treatment trials in CPR patients. However, we were not able to find any preclinical studies exploring the influence of Morphine or Ketamine application before or during CPR on arterial blood pressure blood gas tension, and early survival. Thus, the aim of this experimental study in a rat model of asphyxial cardiac arrest (ACA) was to investigate the influence of pre-treatment with Morphine or Ketamine on hemodynamics, acid-base status, brain damage markers, and early survival as endpoint of the study.

## Methods

### Ethics

The experimental study was approved by the Animal Care and Use Committee of the Astana Medical University, Astana, The Republic of Kazakhstan. The experimental procedures were performed according to the Guide for the Care and Use of Laboratory Animals, Eighth Edition, 2011 formulated by National Academy of Sciences, the United States of America.

### Animal housing

A total of 42 adult male Wistar rats, weighing 350–400 g were pushed from Astana Laboratory Animal Center, Astana, the Republic of Kazakhstan. All experiments were performed in the Experimental Animal Center, Astana Medical University, Astana, the Republic of Kazakhstan. The rats were housed in stainless steel cages (5 rats/cage) at conventional controlled conditions (temperature 25 ± 2 °C; relative humidity 50 ± 10%; 12 h light: dark cycle) and had a free access to standard laboratory food and tap water. The rats acclimated to the condition for 1 week prior to experiments and fasted overnight prior to surgery, with free access to water.

### Animal instrumentation

Under anaesthesia with Thiopental Sodium (Kiev Medpeparat, Ukraine) 60 mg/kg, i.p., rats were tracheostomized with a stainless-steel tracheal tube, connected to a small animal ventilator (TOPO Dual mode ventilator, Kent Scientific Corp, USA) and mechanically ventilated with a tidal volume of 8 ml/kg using room air. A 24G central venous catheter (Arrow) was inserted into the right femoral vein for drug administration and blood sampling. A 22G catheter (22G venflon, BD, Sweden) was inserted into the right femoral artery connected to pressure transducer for continuous blood pressure monitoring using Dash 5000, GE Healthcare, USA. Average time for the instrumentation was about 10 min. At the end of the instrumentation the rats were given vecuronium bromide (Pfizer, USA) 2 mg/kg, i.v.

### Animal randomization

After instrumentation and following a 10 min pause, by means of sealed envelopes the rats were randomly assigned to 3 groups: 1). Morphine group (*n* = 14), where the rats were given i.v. Morphine (Chimfarm Santo, Kazakhstan), 5 mg/kg, 10 min before inducing asphyxial cardiac arrest (ACA). 2). Ketamine group, (n = 14), where the rats were given i.v. Ketamine (Farmac, Ukraine) 40 mg/kg 10 min before ACA, 3). Control group (n = 14), where the rats were given an equal quantity of NaCl 0.9% 10 min before ACA.

### Induction of ACA

**Fig. 1:**
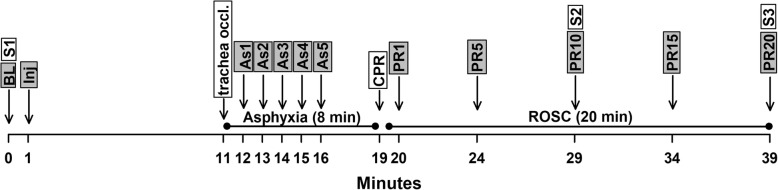
Experimental protocol timeline. (BL) - baseline; (Inj) - injection of study drug or saline; (As1, 2, 3, 4, or 5) - asphyxia at 1, 2, 3, 4, or 5 min; (PR1, 5, 10, 15, or 20) - post-resuscitation at 1, 5, 10, 15, or 20 min; (CPR) - cardiopulmonary resuscitation; (ROSC) - return of spontaneous circulation; (S1) - blood sampling at BL for blood gases and biochemical markers; (S2) - blood sampling at PR10 for blood gases; (S3) - blood and tissue sampling at PR20 (endpoint)

ACA was induced by corking of the tracheal tube for 8 min (Fig. 1), and defined as a mean arterial pressure (MAP) < 20 mmHg. Cardio-pulmonary resuscitation (CPR) was initiated by an i.v. injection of epinephrine (0.02 mg/kg), followed by mechanical ventilation (80 breaths/min) using room air, and manual thoracic compressions (180 compressions/min). Restoration of spontaneous circulation (ROSC) was defined as a return of MAP > 60 mmHg. Ventilation was maintained until spontaneous breathing began. Core temperature (rectal) was kept between 36.5 °C and 37.5 °C using a heating pad. Arterial blood samples were taken at baseline, and 10 min after start of CPR. MAP was recorded at baseline, after i.v. injection of the study drugs or saline, at 1, 2, 3, 4, 5 min after induction of ACA and at 1, 5, 10, 15, 20 min in the post-resuscitation period. All surviving rats were euthanized with 180 mg/kg i.v. of Thiopental Sodium (Kiev Medpeparat, Ukraine) at the end of study.

### Measurement of biochemical markers

Blood samples were centrifuged, plasma were aliquoted and snap frozen at − 70C. Right after euthanasia brain was retrieved and brain tissue samples were snap frozen at − 70 °C. All samples were stored at -70 °C until analysis. Levels of neuron specific enolase (NSE), and s100 calcium binding protein B (s100B) were measured in plasma samples which were collected at baseline and at 10 min in the post-resuscitation period (*n* = 7). Level of caspase-3 (CS-3) was measured in brain tissue samples from the surviving rats at the end of the experimental protocol, 20 min in the post-resuscitation period (n = 7). CS-3 level was normalized to the protein concentration in the brain tissue samples and results presented as a concentration per mg protein. All analysis were performed using Enzyme Linked Immunosorbent Assay (ELISA) kits provided by MyBioSource Inc. (San Diego, CA, USA). Protein content in the brain tissue samples was determined using Quick start Bradford protein assay from Bio-Rad (Hercules, CA, USA).

### Statistical analysis

As we were not able to find any experimental study of Morphine or Ketamine application for animals with asphyxial cardiac arrest, for our study we calculated the sample size based on data from the research study of Endoh H, et al. [22]. In the experimental study with rats exposed to hypoxic gas (5% oxygen, 95% N2) for 70 min, approximately 90% rats survived in the Morphine (5 mg/kg) pre-treated group, and 40% survived in the control group. At 5% of significance level and 80% power, sample size will be pooled prevalence = 0.4 + 0.9/2 = 0.65.

Sample Size = 2 (1.96 + 0.842)^2^ × 0.65 (1–0.65)/(− 0.5)^2^ = 14.26.

based on formula of sample size = 2 (Zα/2 + Zβ)^2^ × P (1 − P)/(p1 − p2)^2^.

where Zα/2 = Z0.05/2 = Z 0.025 = 1.96 (From Z table) at type 1 error of 5% and.

Zβ = Z0.20 = 0.842 (From Z table) at 80% power.

p1 − p2 = Difference in proportion of events in two groups P = Pooled prevalence = (prevalence in case group [p1] + prevalence in the control group [p2])/2.

Data were analyzed and presented using SigmaPlot statistical software version 13.0 (Systat Software Inc., San Jose, CA, USA). Data were tested for normal distribution with Shapiro-Wilks test. Differences in values between groups were analyzed using one-way ANOVA on ranks. If significant differences were found, all pairwise multiple comparison procedures using Dunn’s method was applied to compare values between groups. Blood gas data and data of biochemical markers made after 10 min in the post-resuscitation period vs. corresponding baseline levels within each group were compared using a paired *t*-test. Survival was tested using log-rank Kaplan-Meier test. When significant differences were found, all pairwise multiple comparison procedures were tested using Holm-Sidak method to compare differences between groups. Differences were considered significant at *p* < 0.05.

## Results

**Fig. 2:**
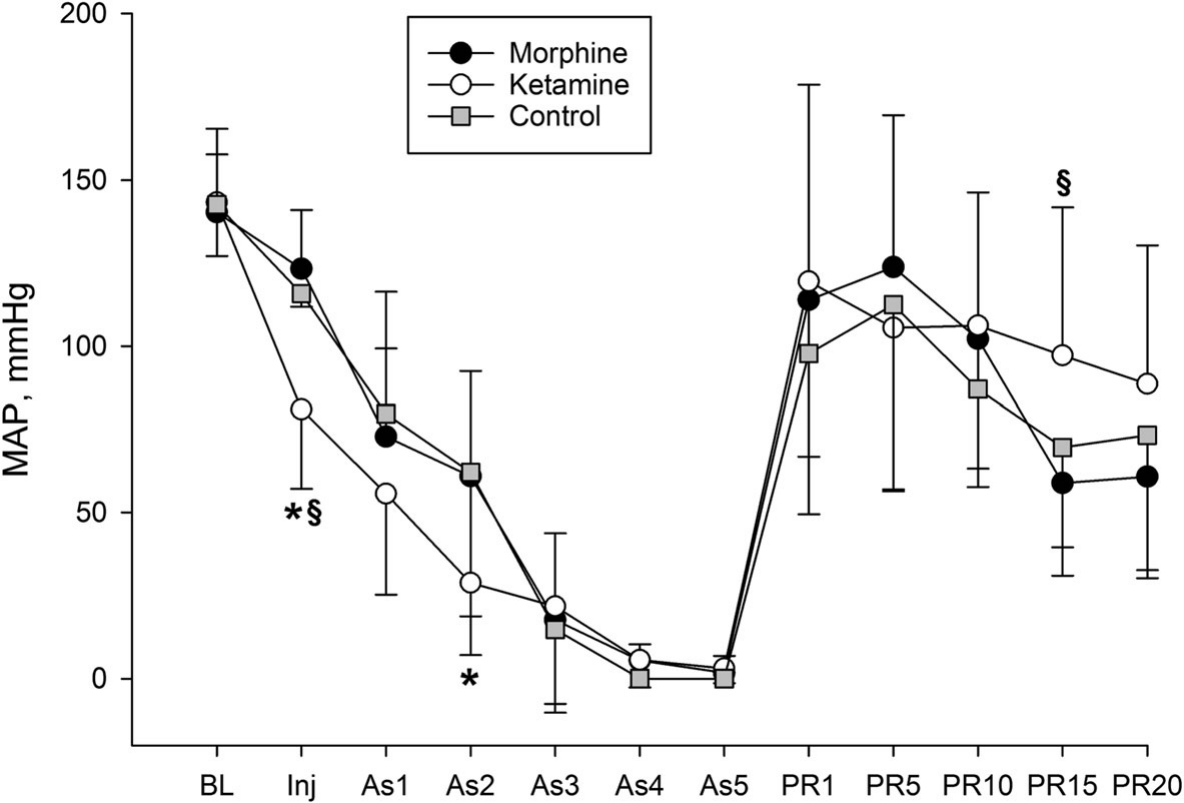
Mean Arterial Pressure (MAP) recorded at baseline (BL), injection of study drug or saline (Inj), asphyxia at 1, 2, 3, 4 or 5 min (As1, 2, 3, 4 or 5), post resuscitation at 1, 5, 10, 15 or 20 min (PR1, 5, 10, 15 or 20). *p < 0.05 vs. control group, ^§^p < 0.05 vs. morphine group. Data presented as mean ± SD, *n* = 14

**Fig. 3:**
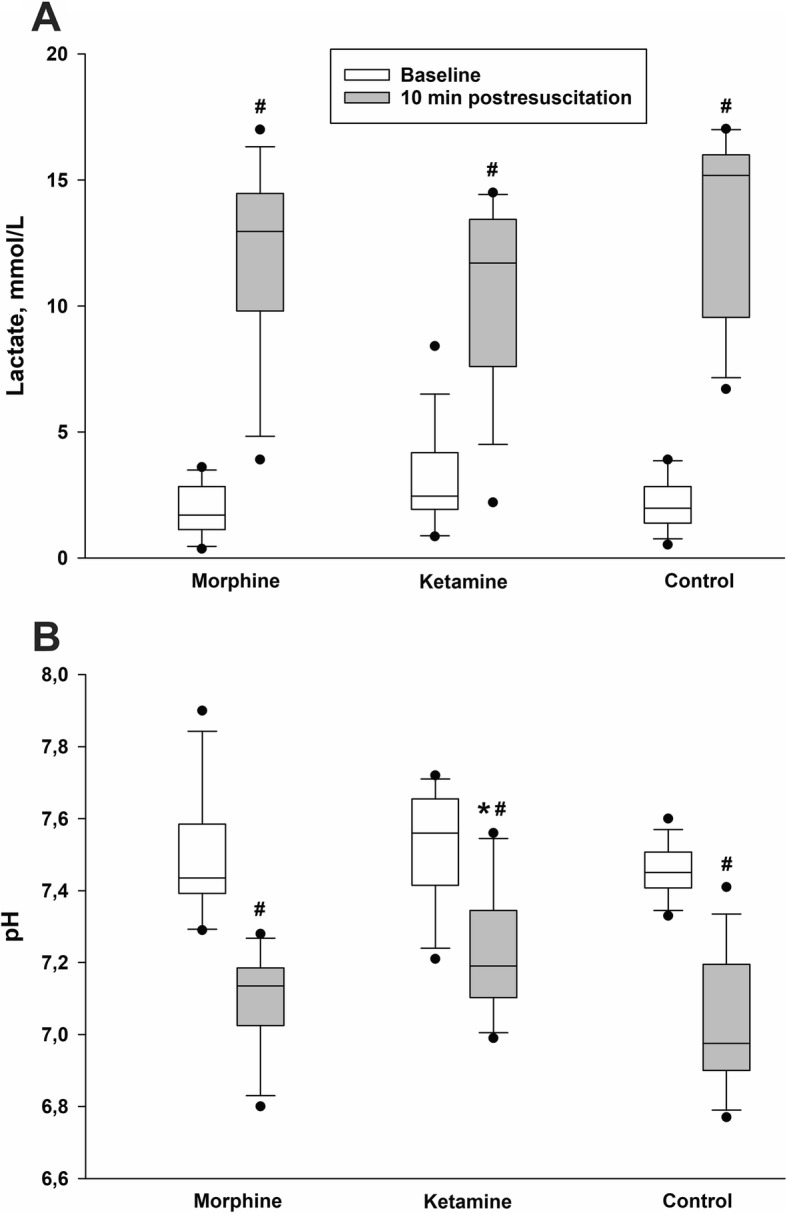
Serum lactate level (**a**) and accumulation of H^+^ in the blood (**b**) measured at baseline and at 10 min in post resuscitation period. Data presented as median 25th and 75th percentiles (vertical boxes with a median line), 10th and 90th percentiles (error bars) and 5th and 95th percentiles (black dots) where # p < 0.05 vs. baseline levels and * p < 0.05 vs. control group

**Fig. 4:**
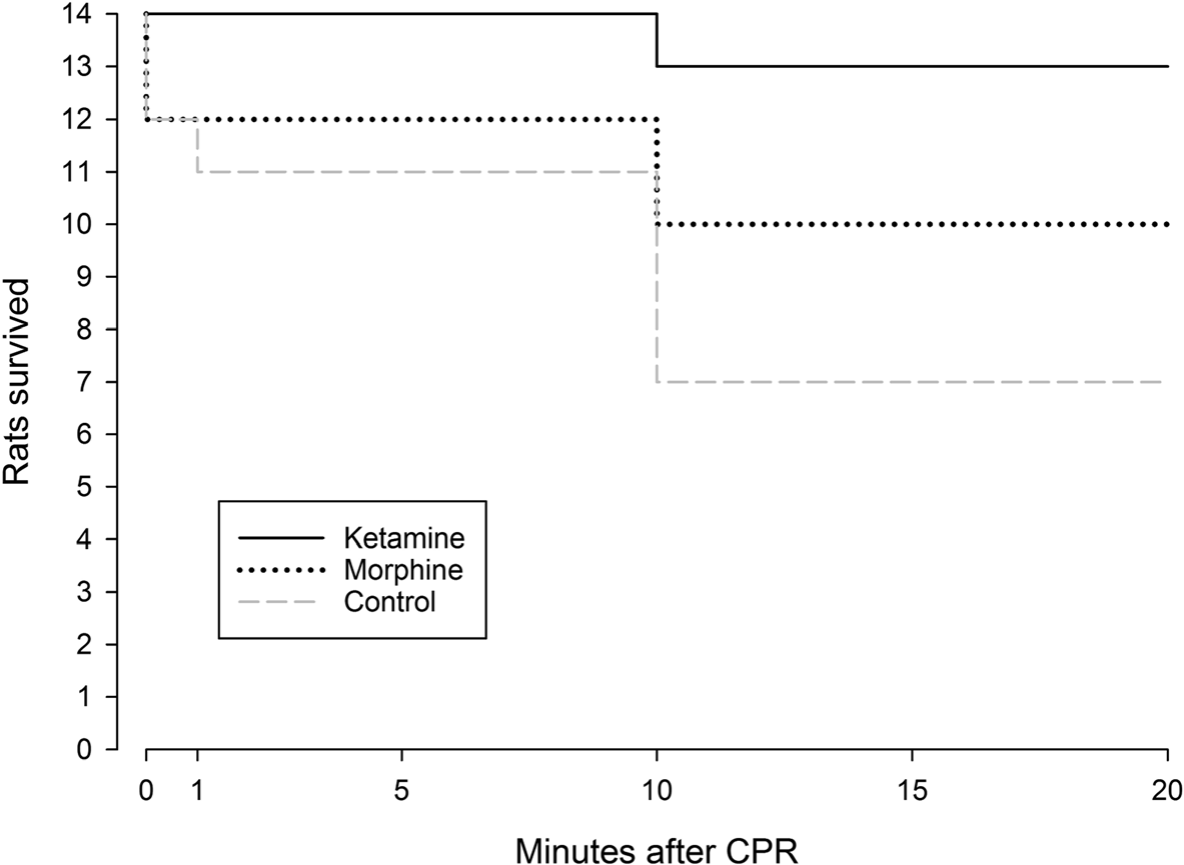
Cumulative survival of the rats at 20 min after CPR, *p* = 0.035 in ketamine vs. control group, n = 14

At baseline (BL), no significant differences in MAP, blood gases, or acid-base status were found between groups (Figs. 2-3, Table 1). As depicted in Fig. 2, pre-treatment of rats with Ketamine resulted in a significant reduction in MAP when compared to rats pre-treated with Morphine or saline. During the first 3 min of asphyxia MAP consistently decreased in all groups resulting in ACA which eventually took place in all animals when invasive MAP dropped below 20 mmHg and remained around zero following 5 min of asphyxia (Fig. 2). Within 3 min, after start of CPR the rats in all groups had ROSC (no differences between the groups) with regained an invasive MAP > 100 mmHg (Fig. 2). At 15 min in post-resuscitation period rats in the Ketamine group had MAP at a significantly higher level compared to rats in the Morphine group, however at the 20 min post-resuscitation no significant difference in MAP between groups was observed. All groups had significantly increased plasma lactate level (10.5–13 mmol/l) compared to their baseline levels (1.8–3 mmol/l) (Fig. 3:A). No significant difference in plasma lactate level between groups was observed. All groups had significantly lower pH value 10 min post-resuscitation (7.0–7.2) compared to intragroup baseline (7.4–7.5) (Fig. 3:B). In addition, rats in the Ketamine group had significantly lower accumulation of hydrogen ions in blood as compared to rats in the Control group (Fig. 3:B). All rats in the study were ventilated with room air only during the whole experiment. Only one rat in the Ketamine treated group died during the post-resuscitation period (death occurred between 10 and 20 min after ROSC). In contrast to the Ketamine group, significantly higher mortality (*p* = 0.035) was observed in the Control group (Fig. 4), where 7 of 14 rats had not survived 20 min after ROSC, 3 of them had died during the first 10 min of the post-resuscitation period. In the Morphine treated group, totally 4 of 14 rats died within 20 min of the post-resuscitation period, 2 of them had died during the first 10 min after ROSC. No differences in blood gases variables (such as SaO_2_, PaO_2_, PaCO_2_) and acid-base status variables (HCO_3_ and BE) were observed between groups. All above mentioned variables except for PaCO_2_ were significantly decreased compared to intragroup baseline (Table 1). Biochemical analysis of plasma concentration of NSE (Table 2) and s100 calcium binding protein B (data not shown) as well as an analysis of caspase-3 levels in the brain tissue (Table 2) did not reveal any differences between the study groups. NSE level was significantly increased after 20 min of post-resuscitation period compared to baseline in all three groups (Table 2).

**Table 1:** Blood gases (mm Hg) and acid base variables measured at the baseline (BL) and at 10 min after asphyxia in post-resuscitation period (10 PR), p between the groups. Data presented as mean ± SD

	morphine		ketamine		control		p
	BL	10 PR	BL	10 PR	BL	10 PR	
SaO_2_	94.1 ± 5.2	66.7 ± 27.3	93.7 ± 7.4	74.1 ± 21.1	93.4 ± 4.3	68.3 ± 23.5	ns
PaO_2_	90.8 ± 16.5	63 ± 21.7	105 ± 18.9	70.8 ± 33.8	88.7 ± 18.5	64.9 ± 25.8	ns
PaCO_2_	30.4 ± 12.3	39.1 ± 26.9	28.3 ± 13.6	22.6 ± 12.3	34.3 ± 12.7	36.2 ± 22.6	ns
HCO_3_	24.4 ± 4.6	10 ± 3.1	24.3 ± 5.1	10.4 ± 3.4	24.4 ± 4.2	10.3 ± 3.7	ns
BE	6 ± 1.7	−19.8 ± 2.3	6.8 ± 2.2	−18.6 ± 4.5	5.2 ± 2.8	−19.9 ± 4.4	ns

**Table 2:** Biochemical analysis of neuron specific enolase (NSE) plasma concentration in ng/ml and caspase-3 (CS-3) levels in the brain tissue of the rats in ng/ml/mg protein, measured at the baseline (BL) and at 20 min after asphyxia in post-resuscitation period (20 PR), p between the groups. Data presented as mean ± SD

	morphine		ketamine		control		p
	BL	20 PR	BL	20 PR	BL	20 PR	
NSE	1.2 ± 0.1	1.8 ± 0.5	0.9 ± 0.1	3.5 ± 2.2	1.1 ± 0.2	2.4 ± 0.5	ns
CS-3	–	0.14 ± 0.02	–	0.16 ± 0.06	–	0.17 ± 0.05	ns

## Discussion

The main finding of the present study was that pre-treatment of rats with Ketamine significantly increased early survival after 8 min of asphyxia and followed by 5 min cardiac arrest. Pre-treatment of rats with Morphine or Ketamine did not result in any significant changes of hemodynamic and biochemical markers of brain damage. However, in the Ketamine pre-treated group rats had significantly better pH levels as compared to the Control group.

The rat model of ACA used in our study was developed by Katz L and co-authors in 1995 [29]. In their study, the authors presented the reproducible and well-documented outcome model of asphyxial cardiac arrest in rats [29]. In this model, rats were anaesthetized with 3% Halothane and 60:40% Nitrous Oxide (N_2_O):oxygen (0_2_) and paralyzed with Vecuronium 2 mg/kg i. v. Followed apneic asphyxia for 8 min led to the well reproducible cessation of blood circulation at 3–4 min of apnea and cardiac arrest for 4–5 min. Survival to 72 h after ACA was achieved in 9 of 10 rats (90%) in the study. All survived rats had mild neurologic deficit scores that primarily were due to hind-leg spastic paralysis. However, the paralysis was due to insertion of arterial and venous catheter in the femoral vessels with following ligation and cessation of blood circulation in the leg [29]. In contrast to the «classical» model, in our study the rats were anesthetized with Thiopental. Recently it was demonstrated that Thiopental significantly depresses both cardiac and respiratory function, making cardiac pulmonary resuscitation in rats more difficult [28]. Definitely, application of Thiopental anaesthesia and absence of pre- and post-100% oxygenation in our study resulted in 50% mortality in the Control group (Fig. 4). The high mortality in our study makes our experimental model more relevant to clinical situations where early survival after in-hospital cardiac arrest recently was demonstrated approximately to be 50% for all patients with well-documented cardiac rhythms [30]. Despite on basic anaesthesia with Thiopental, pre-treatment with Ketamine dramatically increased early survival (93%) in the rats (Fig. 5). The results are supported by an earlier finding of Reid KH et al. [28], who demonstrated a successful restoration of cardiac function after CA in 81% of rats anesthetized with Ketamine versus ROSC in 39% rats under Thiopental anaesthesia. In our opinion, high early survival (90%) in the «classical» model of Katz L and co-authors [29] might also be related to NMDA blockade by N_2_O. Meanwhile, two experimental studies testing effects of two NMDA antagonist, MK-801 and GPI-3000 demonstrated no improvement of survival rate and brain outcome after CA in a dog model [31, 32]. These studies did not suggest any mechanisms for the negative results, but they apparently have contributed to a lack of interest for testing NMDA blockade in CA for years. However, new published experimental data demonstrates that pre-treatment of zebrafish with Ketamine protects against cardiac arrest-induced brain injury by inhibiting Ca^2+^ wave propagation, which consequently improves survival rate [33]. More recently, a study of the effects of using the noncompetitive NMDA antagonist Ifenprodil demonstrated a significant reduction of brain edema following CA in rats [34]. In this study, i.v. injection of Ifenprodil caused a significant reduction of MAP before CA and much more stable hemodynamic after CA as compared with saline treated animals [34]. Consistent with these findings [34], in our study the rats pre-treated with Ketamine demonstrated a significant reduction of MAP right after i.v. injection, but showed a relatively stable hemodynamic after CA. Summarising the above, most likely that the sympathomimetic effects of Ketamine together with subsequent improvement in pH levels of rats are the main cause for the significant increment of early survival in our study. As it is not possible to apply cardiac arrest to animal without any anaesthesia (main limitation of all experimental models of cardiac arrest), the sympathomimetic effects and possible neuroprotective features of Ketamine [35] should be tested in patients with cardiac arrest. Additional topic for possible clinical research of Ketamine as well as Morphine could be their analgesic effects as vigorous thoracic compression with possible trauma of the ribs may lead to severe pain and stress reactions in patients surviving CPR.

In an experimental model with rats exposed to hypoxic gas (5% 0_2_, 95% N_2_) for 70 min, all seven rats in the Naloxone pre-treated group died at the end of the experiments while only one out of seven rats died in the Morphine (5 mg/kg) pre-treated group, and five of seven rats died in the control group [22]. The results were very similar to previously published finding obtained from mice in the same model [21]. Interestingly, pre-treatment with Morphine in these studies significantly attenuated MAP and enhanced hypoxic ventilatory depression but, nevertheless, improved hypoxic survival [21, 22]. In our experiments where the rats were exposed to 8 min anoxia, pre-treatment with Morphine resulted in non-significant attenuation of MAP (Fig. 2) and non-significant positive trend in survival (Fig. 4). We were not able to find any publications looking at pre-treatment with Morphine and survival rate in animals after cardiac arrest. However, two recent retrospective studies demonstrated that patients who were treated with opioids before or during CA had a statistically significantly higher survival rate [36] and much better neurological outcome [37] compared to untreated patients.

The rationale for analyzing plasma levels of S-100B protein and NSE in this study was their different distribution within the white (S100B protein) and grey (NSE) matter of the brain, and the fact that both of them are extensively involved in the pathogenesis of anoxial brain damage [38]. S100 B protein is an intracellular calcium-binding dimer that has a molecular weight of 21 kDa and a half-life of two hours. Thanks to the low molecular weight, S100 B easily crosses the blood-brain barrier and ends up rapidly in the systemic circulation. In this study, we did not find any changes in the plasma level of S100 B, and therefore data is not presented. NSE is a neuronal isoform of the glycolytic enzyme enolase that has a molecular weight of 78 kDa and a twenty-four hours half-life. Further, NSE is extensively involved in glucose metabolism in the neurons and can be detected only in neuronal and neuroendocrine tissues. Due to this organ specificity, concentration of NSE in blood is often elevated because of relative rapid and massive neuronal destruction. In the present study, plasma levels of NSE were found to be slightly increased at 20 min after cardiac arrest in all groups compared to the baseline measurement (Table 2), but the levels did not exceed the normal range of NSE in blood, considered to be ≤15 ng/ml. Caspase-3 is involved in the early stage of apoptosis and is currently considered to be the hallmark of irreversible cell death [10]. As depicted in Table 2, tissue levels of caspase-3 remained low in all study groups and no significant differences between groups were found. When summarising all the biochemical findings in the study, we can conclude that independent of pre-treatment, there was an absence of biochemical signs of apoptosis in the rats at 20 min after ACA. Our results find support in a previous study [39] of post-mortem adult rat brains, which demonstrated absence of autolytic damages in the ultrastructure of cerebral neurons during the first 6 h after warm asphyxial cardiac arrest. Interestingly, in the referred study, the activation of caspase-3 was observed in a significant number of neurons of the cerebellum and neocortex only after 9 h following asphyxial cardiac arrest [39].

Our study has certain limitations. We did not perform any monitoring of cardiac output in the rats and therefore no cardio depressive effect of Morphine or Ketamine after ACA was elucidated. However, as arterial blood pressure and accumulation of lactate were not significantly different between the groups we may speculate whether the negative influence of Morphine or Ketamine on heart function were clinically irrelevant. We did not measure brain oxygen demand in our rats, and therefore the influence of Morphine or Ketamine on oxygen consumption remains unsettled. Finally, rapid intracellular accumulation of both Na^+^ and Ca^2+^ during anoxia might have contributed to development of brain edema, thus further research is warranted to elucidate the influence of Morphine or Ketamine on the development of cerebral edema after CA.

## Conclusions

Pre-treatment with Ketamine before ACA significantly improved early survival and attenuated alterations in pH after ROSC when compared to placebo control rats. Additionally, a positive trend for increased survival was also observed in the rats pre-treated with Morphine. Further experimental studies are needed to elucidate effects of Ketamine and/or Morphine on long-term survival and neurological outcome after ACA.

## Data Availability

The data that support the findings of this study in form of Excel files are available from the corresponding author.

## Abbreviations

ACA: asphyxial cardiac arrest
BL: baseline
CA: cardiac arrest
CPR: Cardiopulmonary resuscitation
CS-3: Caspase-3
ELISA: Enzyme Linked Immunosorbent Assay
MAP: mean arterial pressure
NMDA: N-methyl-D-aspartate
NSE: neuron specific enolase
ROSC: Return of spontaneous circulation
s100B: s100 calcium binding protein B
